# HLH and spinal neurofibroma: a single case report in a patient with DiGeorge syndrome

**DOI:** 10.3389/fonc.2026.1771627

**Published:** 2026-03-20

**Authors:** Ramona Tallone, Concetta Micalizzi, Maura Faraci, Patrizia De Marco, Valeria Capra, Andrea Beccaria, Virginia Livellara, Elena Arkhangelskaya, Antonia Ramaglia, Patrizia Ronchetto, Marzia Ognibene, Maria Bono, Monica Muraca, Valerio Gaetano Vellone, Chiara Rebuffi, Stefania Marcenaro, Lisa Pelanconi, Riccardo Haupt, Carlo Dufour, Marina Francesca Strati, Silvia Merlo

**Affiliations:** 1 Diagnosis, Observation, and Prevention after Oncological therapy (D.O.P.O.) Clinic, Department of Pediatric Hematology and Oncology, Istituto di Ricovero e Cura a Carattere Scientifico (IRCCS), Istituto Giannina Gaslini, Genoa, Italy; 2 Hematology Unit, Department of Pediatric Hematology and Oncology, Istituto di Ricovero e Cura a Carattere Scientifico (IRCCS), Istituto Giannina Gaslini, Genoa, Italy; 3 Hematopoietic Stem Cell Transplantation Unit, Istituto di Ricovero e Cura a Carattere Scientifico (IRCCS), Istituto Giannina Gaslini, Genoa, Italy; 4 Medical Genetics Unit, Istituto di Ricovero e Cura a Carattere Scientifico (IRCCS), Istituto Giannina Gaslini, Genoa, Italy; 5 Genomics and Clinical Genetics, Istituto di Ricovero e Cura a Carattere Scientifico (IRCCS), Istituto Giannina Gaslini, Genoa, Italy; 6 Oncology Unit, Department of Pediatric Hematology and Oncology, Istituto di Ricovero e Cura a Carattere Scientifico (IRCCS), Istituto Giannina Gaslini, Genoa, Italy; 7 Pediatric Radiology, Istituto di Ricovero e Cura a Carattere Scientifico (IRCCS), Istituto Giannina Gaslini, Genoa, Italy; 8 Neuroradiology, Istituto di Ricovero e Cura a Carattere Scientifico (IRCCS), Istituto Giannina Gaslini, Genoa, Italy; 9 Laboratory of Human Genetics, Istituto di Ricovero e Cura a Carattere Scientifico (IRCCS), Istituto Giannina Gaslini, Genoa, Italy; 10 Unità Operativa Complessa (UOC) of Pathology, Istituto di Ricovero e Cura a Carattere Scientifico (IRCCS) Istituto Giannina Gaslini, Genoa, Italy; 11 Scientific Direction, Istituto di Ricovero e Cura a Carattere Scientifico (IRCCS), Istituto Giannina Gaslini, Genoa, Italy; 12 Molecular Diagnostics Unit of Hospital Pathological Anatomy, Istituto di Ricovero e Cura a Carattere Scientifico (IRCCS) Metropolitan San Martino Hospital, Genoa, Italy; 13 Department of Neuroscience, Rehabilitation, Ophthalmology, Genetics and Maternal-Infantile Sciences, University of Genoa, Genoa, Italy

**Keywords:** 22q11.2 deletion, DiGeorge syndrome, haploinsufficiency, hemophagocytic lymphohistiocytosis (HLH), immunodeficiency, LZTR1, neurofibroma

## Abstract

**Background:**

DiGeorge syndrome is a rare genetic disorder with variable phenotypic and immunologic features associated to an increased risk of malignancy. Hemophagocytic lymphohistiocytosis (HLH) is a rare hyperinflammatory condition that can be triggered by infectious complications in immunodeficient patients like those with DGS.

**Case:**

We report the case of a 20-year-old female with intellectual and motor disabilities who was diagnosed with non-familial HLH at age of 12 months and underwent allogeneic hematopoietic stem cell transplantation due to early disease reactivation. Despite resolution of HLH and post-transplant GVHD, the patient’s psychomotor delay persisted, and dysmorphic features became more pronounced. A an array-CGH on her fibroblasts was performed and identified a partial *de novo* deletion on chromosome 22, consistent with DiGeorge Syndrome (DGS). At 15 years, imaging performed because of persistent and worsening back pain, revealed an intraneural/intraradicular atypical neurofibroma which was partially resected leading to symptoms disappearance.

**Conclusion:**

This is the first report of a case of DGS with early onset HLH and late occurrence of a neoplasm of uncertain biological potential. Careful clinical monitoring is essential due to the variability in clinical manifestations and level of immune alteration.

## Introduction

The occurrence in the same subject of genetic disorders and clinical conditions as congenital malformations, occurrence of rare tumors, or tumors occurring at unusual age or site may be of scientific interest since they can help in the identifications of genetic pathways associated to these conditions.

In this context, we report what we believe to be the first documented case of an association between DiGeorge syndrome, hemophagocytic lymphohistiocytosis (HLH), and an intraneural/intraradicular neurofibroma, and we propose hypotheses regarding the possible underlying pathogenic mechanisms.

## Case presentation

**Figure 1:**
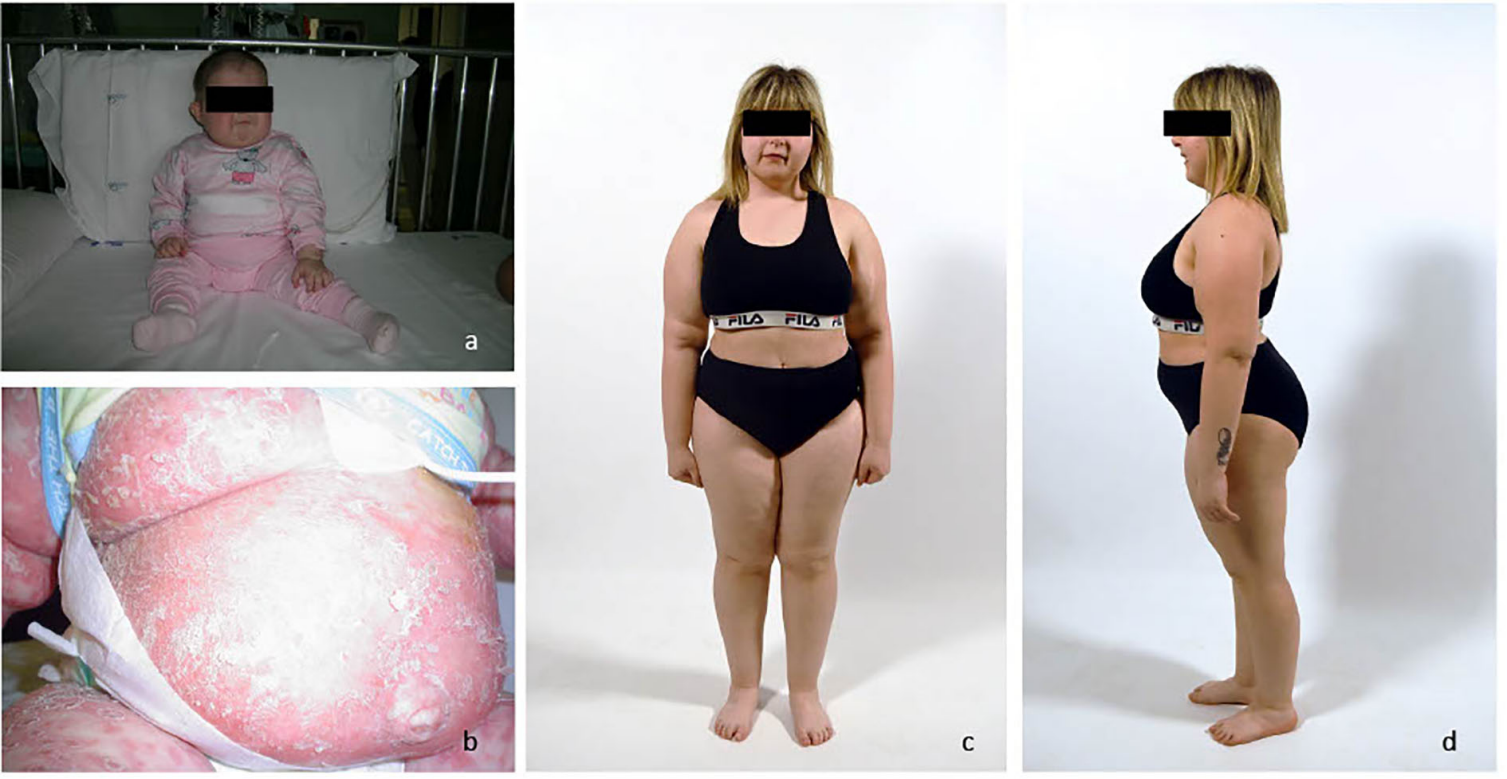
**(A)** image of the patient at 6 months of age; **(B)** image of severe cutaneous GVHD; **(C, D)** current images of the patient.

A female infant born to non-consanguineous parents, appropriate for gestational age and with an unremarkable perinatal history, at 8 months was noted to have psychomotor delay characterized by hypotonia and muscle atrophy (**Figure 1A**). At 12 months, she presented with intermittent fever, elevated liver enzymes (aspartate and alanine aminotransferase AST/ALT 464/501 U/L), two-lineage cytopenia (hemoglobin 7.1 g/dL, platelets 79,000/mm³), hypertriglyceridemia (419 mg/dL), increased ferritin (512 ng/mL), hypofibrinogenemia (146 mg/dL), hypoalbuminemia (2.500 mg/dL), and hepatosplenomegaly. Serological tests for toxoplasmosis, parvovirus, cytomegalovirus (including CMV-DNA), and herpes virus (including EBV-DNA) were negative. Lymphocyte subpopulation assays showed normal value of T and NK cells, with normal perforin expression, normal NK cell degranulation and cytotoxic activity (details on results and references in Supplementary Material 1). Genetic testing for perforin and hMunc 13–4 genes were normal. Accordingly, a clinical and laboratoristic diagnosis of CNS positive, non-familial Hemophagocytic lymphohistiocytosis (HLH) was made, as of the Histiocyte Society criteria (1) Details in **Supplementary Table 1**].

Brain MRI revealed small gliotic-malacic lesions in the right *corona radiata* and basal nucleus, likely due to systemic inflammation. Additionally, bilateral semicircular canal dysplasia, reduced volume of the pons and cerebellum, and simplified cortical gyration were observed.

Treatment with dexamethasone, cyclosporine, and etoposide was started along with intrathecal methotrexate (MTX), according to the HLH 2004 protocol. Clinical and laboratory remission was achieved but lasted only two months. During continuation therapy, signs of macrophage reactivation re-emerged [fever, elevated liver enzymes (AST/ALT 611/1448 U/l), increased ferritin (618 ng/ml), hypertriglyceridemia (425 mg/dl), hypofibrinogenemia (127 mg/dL), and hypoalbuminemia (2352 mg/dL)], necessitating the re-initiation of the induction phase.

After achievement of a second remission, the patient was treated with allogeneic HSCT from a matched unrelated donor conditioned with fludarabine (150 mg/m²) and melphalan (140 mg/m²) without total body irradiation. The graft versus host disease (GVHD) prophylaxis was based on cyclosporine, anti-lymphocyte globulin and MTX. Three months after HSCT, the patient developed grade III cutaneous GvHD, according to the NIH classification, characterized by scaly and likenoid lesions on 100% of body skin, which was treated with extracorporeal photophoresis and low doses of cyclophosphamide (**Figure 1B**). Due to recurrent flares during tapering attempts, cyclophosphamide was continued for an extended period. Immunosuppressive therapy was eventually discontinued at 5 years of age, three years after HSCT.

Despite resolution of HLH and GVHD, the patient’s psychomotor delay persisted, and dysmorphic features (short stature, microcephaly, hypertelorism, chin hypoplasia, and small, low-set ears) became more pronounced (**Figures 1C, D**). Consequently, an array-CGH analysis was performed. Genomic DNA was obtained from proband’s lymphoblastoid cell lines and parents’ peripheral blood. Array-CGH was performed (Sureprint G3 Human CGH Microarray Kit 8 × 60k) and analyzed by Agilent Cytogenomics 5.2.1.4 software, and genomic positions were reported according to the human genome assembly GRCh37/hg19. A *de novo* deletion (2.5 Mb) on the long arm of chromosome 22 from 18,919,942 (22q11.21) to 21,440,514 (22q11.21) associated with DiGeorge Syndrome (DGS - OMIM 188400) was documented. Two of the three major genes associated to DGS (*CrkL* and *Tbx1*) were mapped in the deletion region. Moreover, a paternally inherited duplication on chromosome 10q26.13 spanning approximately 590 Kb, from 123,297,473 (10q26.13) to 123,887,381 (10q26.13) was also documented. This duplication involves the genes ATE1, NSMCE4A, TACC2 and the fibroblast growth factor receptor-2 *FGFR2* gene (OMIM 176943) which is classified as variant of uncertain significance (VUS).

**Figure 2:**
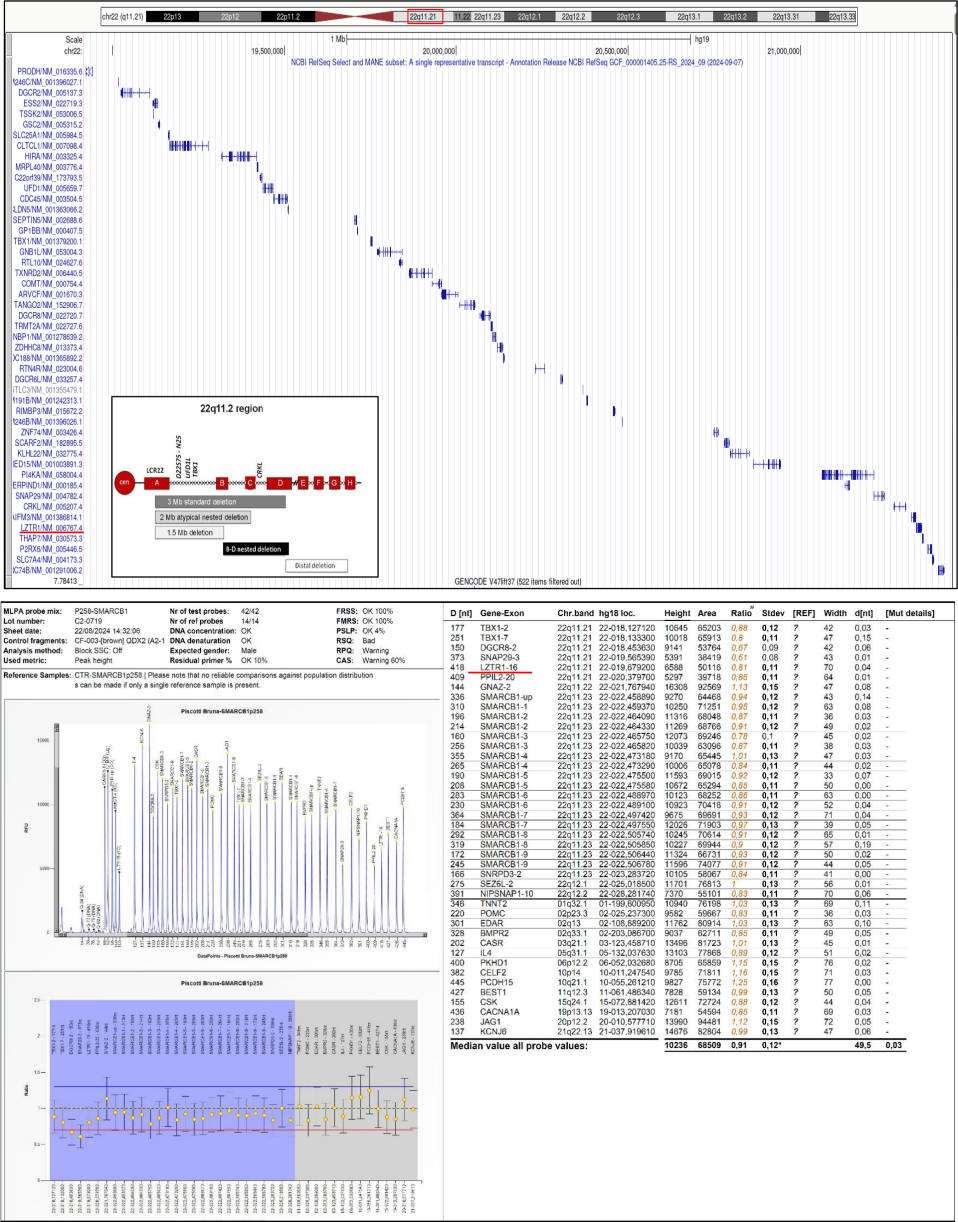
MLPA analysis of the deletion identified by array CGH, highlighting the deletion interval and the mapping of the *Crkl* and *Tbx1* genes. Bottoms: MLPA performed on the tumor tissue; in red, evidence of haploinsufficiency affecting the *LZTR1* gene.

Follow-up was uneventful until 17 years when the patient reported persistent and worsening back pain. A cranial-spinal MRI revealed a bulky right thoracic paravertebral/extra- and intra-foraminal lesion at the T3–T5 level, and the persistence of the previously described peculiar cerebral lesions. Partial neurosurgical excision was performed and pathology documented the presence of intraneural/intraradicular neurofibroma with atypical areas of neurofibromatous neoplasm of uncertain biological potential (ANNUBP - WHO Classification of Soft Tissue and Bone Tumors 2019). Given the constitutional diagnosis of DGS, and the absence of documented cases linking intraneural tumors to this condition, we reviewed the previously performed array-CGH which indeed also documented a haploinsufficiency of the *LZTR1* gene (leucine-zipper-like transcriptional regulator 1), which is associated with susceptibility to Schwannomatosis type 2 and Noonan Syndrome type 10. However, MLPA analysis of the tumor-derived DNA did not show a loss of the second *LZTR1* allele (**Figure 2**). No pathogenic variants were identified in the *NF1* and *NF2* genes. No progression of the residual tumor has been documented after a six-monthly oncological and radiological follow-up, and the patient is well without significant back problems, 36 months after surgery.

## Discussion

To the best of our knowledge, this is the first reported case of DGS who developed HLH followed by a neurofibromatous tumor with atypical features.

HLH is a life-threatening hyperinflammatory syndrome characterized by uncontrolled and excessive immune activation, which is usually suspected in the presence of prolonged fever, hepatosplenomegaly and cytopenia. As in this case, the diagnosis is established when at least five out of the eight HLH-2004 diagnostic criteria of the Hystiocyte Society are fulfilled. [1, 2, **Supplementary Table 1**]. It is categorized into the acquired and the genetic forms. The acquired forms usually occur in older children and adults being associated with infections, cancers, autoimmune disorders. Genetic HLH usually occurs among infants and young children, and is further categorized as: i) familial due to mutations affecting perforin or the granule-dependent cytotoxic activity; ii) secondary to immunodeficiency syndromes which may be either hereditary (e.g. Chediak-Higashi or Griscelli syndrome); or secondary to *de novo* mutations as in DGS (3).

DGS is caused by a heterozygous deletion on chromosome 22q11.2 due to a non-allelic meiotic recombination during spermatogenesis or oogenesis which may include three key genes: *CrkL* (CRK Like Proto-Oncogene, Adaptor Protein), *Tbx1* (T-box protein 1), and *Erk2* (extracellular signal-regulated kinase 2). In our patient (**Figure 2**), only *Tbx1* and *Erk2* were deleted while *CrkL* was not involved in the deletion. *Tbx1* deletions are implicated in cardiac, parathyroid, thymus and facial structure development and thus explain the facial dysmorphisms of our patient. *Erk2*, besides being involved in cellular growth differentiation, proliferation and apoptosis has also a role in immune cell signaling shaping the inflammatory response to infection and cell damage. The normal T and NK cell counts observed in our case are explained by the lack of *CrkL* silencing which is known to be involved in T cell dysfunction and impaired NK cell function (4).

The DGS patients are usually categorized in two phenotypical forms ranging from the complete (severe) sDGS with athymia and severe T-cell immunodeficiency (>1% of cases), to the partial DGS (pDGS) characterized by less severe forms with variable phenotypical characteristics. Currently, there is no definitive genotype-phenotype correlation to explain the clinical or immunological differences (5).

The occurrence of HLH in patients with DGS is rarely reported. After the first report by Arricò in 1999, Bode described a cohort of 63 patients with HLH and various types of primary immunodeficiencies, identifying only four patients (6.3%) with DGS (5–7). With the limits of this report in which we have not documented any specific trigger for HLH onset, we believe that our patient may be classified as with a pDGS in which secondary HLH probably developed because of an abnormal immune response to an unidentified infectious trigger. This fact might be due to a T cell immunodeficiency linked to the *Tbx1* and *CrkL* deletions which together with *Erk2* (normal in our case) are among the three major genes responsible for the DGS phenotype (4) as reported by Zheng.

Patients with DGS are also reported to have also an increased risk for various types of cancers either hematologic or not, but the mechanisms are not fully understood (8). Malignancies often correlate with specific deletions at the distal region of 22q11.2, involving the INI1/SMARCB1 tumor suppressor gene and the critical region gene 5 (DGCR5) which regulates long non-coding RNAs (lncRNAs) important in tumorigenesis (9). Since our patient does not carry mutations in these regions, we can only speculate a potential etiopathogenic role of the haploinsufficiency of *LZTR1* which has been implicated in a wide spectrum of peripheral nervous system neoplasms with wide spectrum of morphologic and biological potential ranging from benign to highly malignant tumors [Belakhoua]. pathologic and nervous system tumors, such as schwannomas gliomas and spinal tumors (10). MLPA testing on tumor DNA of our patient excluded the complete biallelic loss of *LZTR1* which, as suggested by Evans would be lethal to the cell, thereby preventing tumor development (10). Moreover, NGS sequencing of tumor DNA revealed no mutations in the NF2 and NF1 genes, but only alterations in genes involved in DNA repair (e.g., *BRIP1*).

The potential oncogenic role of duplication of genes *ATE1, NSMCE4A, TACC2*, and *FGFR2* on chromosome 10q26.13, still reported as VUS, has not been documented yet, but their potential role cannot be ruled out and deserves future research.

## Conclusions

In conclusion, this case reports the first association between DGS, HLH and late development of intraneural/intraradicular neurofibroma, it highlights the complexity of DGS, and suggests that, despite the relatively low cancer risk, careful clinical monitoring is essential due to the variability in manifestations and potential immune alterations.

## Data Availability

The original contributions presented in the study are included in the article/**Supplementary Material**, further inquiries can be directed to the corresponding author/s.
