# Supplementary material for: HLH and spinal neurofibroma: a single case report in a patient with DiGeorge syndrome
Source: Front Oncol. 2026 Mar 20;16:1771627. doi: 10.3389/fonc.2026.1771627 (PMC13046545; doi:10.3389/fonc.2026.1771627)
Supplement: Supplementary file 1 [file Table1.docx]

**Supplementary Table1:**

**Results of the HLH-2004 diagnostic panel in the reported case**

| **HLH-2004 diagnostic criteria** | **Test performed** | **Our patient** | |
| --- | --- | --- | --- |
| 1. **A molecular identification of an HLH-associated gene mutation diagnosis consistent with HLH**   (eg. PRF1, UNC13D, STX11, STXBP2, Rab27A, SH2D1A, BIRC4, LYST, ITK, SLC7A7, XMEN, HPS) | PRF1  hMunc | Negative  Negative | |
|  |  |  |  |
| 1. **Clinical and laboratory diagnostic criteria for HLH fulfilled (5 out of the 8 criteria below)** | **Reference values** | **Value** | **Consistent with**  **HLH diagnosis** |
| Fever | Normal <36.4-36.9°C | >38 °C | Yes |
| Splenomegaly | 5-6.5 cm (at 6 months) | 7 cm | Yes |
| Cytopenias (at least 2 of 3 lineages in peripheral blood):  Hemoglobin <9 g/L  Platelets <100,000  Neutrophils <1x10^9^ | (12-14 g/L)  (150-400,000)  2.1-6.49 x10^9^/L | 7.1 g/L  79,000  1.9 x10^9^/ | Yes  Yes  No |
| Hypertriglyceridemia and/or hypofibrinogenemia  Triglycerides ≥ 265 mg/dl  Fibrinogen ≤ 1.5 g/L | 30-160 mg/dl  2-4 g/L | 419 mg/dl  1.4 mg/dl | Yes  Yes |
| Hemophagocytosis in bone marrow or spleen or lymph node | Absent | Present | Yes |
| Low or absent NK-cell activity  (according to local laboratory reference) | False | Normal | No |
| Ferritin ≥ 500 mcg/L | 20-200 mcg/l | 512 mcg/L | Yes |
| Soluble CD25 (i.e., soluble IL-2 receptor) ≥ 2400 U/L | Not applicable | Not done | No |
