# Supplementary material for: HLH and spinal neurofibroma: a single case report in a patient with DiGeorge syndrome
Source: Front Oncol. 2026 Mar 20;16:1771627. doi: 10.3389/fonc.2026.1771627 (PMC13046545; doi:10.3389/fonc.2026.1771627)
Supplement: Supplementary file 2 [file Table2.docx]

**Supplementary material 2**

**Values and methods for perforin expression, NK cell degranulation and cytotoxic activity in the reported case**

| **Test** | **Healthy Controls** | **Our patient** |
| --- | --- | --- |
| Perforin (PRF1) in bulk NK cells, mean value in fluorescence intensity (MFI) analysis | 68  47 | 93 |
| CD107a expression PBMCs cultured overnight with IL-2 and stimulated with K562 target cells, % | 50 | 47 |
| Citotoxicity against melanoma FO-1, % | 44 | 41 |

**References**

- Marcenaro S, Gallo F, Martini S, Santoro A, Griffiths GM, Aricó M, Moretta L, Pende D. Analysis of natural killer-cell function in familial hemophagocytic lymphohistiocytosis (FHL): defective CD107a surface expression heralds Munc13-4 defect and discriminates between genetic subtypes of the disease. Blood. 2006 Oct 1;108(7):2316-23. doi: 10.1182/blood-2006-04-015693. Epub 2006 Jun 15.
- Trambas C, Gallo F, Pende D, Marcenaro S, Moretta L, De Fusco C, Santoro A, Notarangelo L, Arico M, Griffiths GM.: [A single amino acid change, A91V, leads to conformational changes that can impair processing to the active form of perforin.](https://urlsand.esvalabs.com/?u=https%3A%2F%2Fpubmed.ncbi.nlm.nih.gov%2F15741215%2F&e=a39aa7eb&h=f0ec756f&f=y&p=y) Blood. 2005 Aug 1;106(3):932-7. doi: 10.1182/blood-[2004-09-3713](callto:2004-09-3713). Epub 2005 Mar 1.
